# Observing Grasping Actions Directed to Emotion-Laden Objects: Effects upon Corticospinal Excitability

**DOI:** 10.3389/fnhum.2016.00434

**Published:** 2016-08-30

**Authors:** Anaelli A. Nogueira-Campos, Ghislain Saunier, Valeria Della-Maggiore, Laura A. S. De Oliveira, Erika C. Rodrigues, Claudia D. Vargas

**Affiliations:** ^1^Department of Physiology, Federal University of Juiz de ForaMinas Gerais, Brazil; ^2^Laboratory of Motor Cognition, Department of Anatomy, Federal University of ParáBelém, Brazil; ^3^IFIBIO Houssay, Department of Physiology and Biophysics, School of Medicine, University of Buenos AiresBuenos Aires, Argentina; ^4^Post-Graduate Program in Rehabilitation Sciences, UnisuamRio de Janeiro, Brazil; ^5^Laboratory of Neurobiology II, Neurobiology Program, Institute of Biophysics Carlos Chagas Filho, Federal University of Rio de JaneiroRio de Janeiro, Brazil; ^6^Instituto de Neurologia Deolindo Couto, Federal University of Rio de JaneiroRio de Janeiro, Brazil

**Keywords:** motor evoked potentials, motor resonance, valence, goal-directed actions, mirror neurons

## Abstract

The motor system is recruited whenever one executes an action as well as when one observes the same action being executed by others. Although it is well established that emotion modulates the motor system, the effect of observing other individuals acting in an emotional context is particularly elusive. The main aim of this study was to investigate the effect induced by the observation of grasping directed to emotion-laden objects upon corticospinal excitability (CSE). Participants classified video-clips depicting the right-hand of an actor grasping emotion-laden objects. Twenty video-clips differing in terms of valence but balanced in arousal level were selected. Motor evoked potentials (MEPs) were then recorded from the first *dorsal interosseous* using transcranial magnetic stimulation (TMS) while the participants observed the selected emotional video-clips. During the video-clip presentation, TMS pulses were randomly applied at one of two different time points of grasping: (1) maximum grip aperture, and (2) object contact time. CSE was higher during the observation of grasping directed to unpleasant objects compared to pleasant ones. These results indicate that when someone observes an action of grasping directed to emotion-laden objects, the effect of the object valence promotes a specific modulation over the motor system.

## Introduction

One individual's perception of another individual's action and the response this causes in the brain are tightly linked phenomena. The neurophysiological basis of this phenomenon is thought to be based on mirror neurons discovered in the fronto-parietal network, including the premotor cortex, and the intraparietal sulcus (di Pellegrino et al., 1992; Gallese et al., 1996; Rizzolatti et al., 1996; Hari et al., 1998; Buccino et al., 2001; Rizzolatti and Craighero, 2004; Fogassi et al., 2005; Rizzolatti and Sinigaglia, 2010, for review). Mirror neurons are recruited when someone observes an action performed by others and when he/she executes the same action (for review Blakemore and Decety, 2001; Rizzolatti and Craighero, 2004; Rizzolatti, 2005; Fabbri-Destro and Rizzolatti, 2008; Keysers and Fadiga, 2008; Rizzolatti and Sinigaglia, 2010; Sinigaglia and Rizzolatti, 2011). Neurons with mirror-like properties have recently been described in a broader action-perception network involving the primary motor and somatosensory cortices as well as regions related to memory and emotional processing (Mukamel et al., 2010; Molenberghs et al., 2012; Fogassi and Simone, 2013).

Such a vast action-perception network attests to its crucial role in coding others' actions in the brain (Fadiga et al., 1995; Calvo-Merino et al., 2005), in recognizing their meaning (Avenanti et al., 2005; Rossi et al., 2008; Akitsuki and Decety, 2009; Borgomaneri et al., 2012), predicting their consequences (Kilner et al., 2004; Aglioti et al., 2008; Fontana et al., 2012) as well as their intentions (Becchio et al., 2012; Sartori et al., 2012). Furthermore, there is robust evidence that the observer's motor system codes the expected temporal adjustments when the grasping unfolds over time (Gangitano et al., 2004), suggesting a perfect matching between action observation, and its execution (Gueugneau et al., 2015; Mc Cabe et al., 2015). Thus, motor representations activated by observed actions might allow the anticipation and the processing of the meaning implied in such actions (Umiltà et al., 2001; Urgesi et al., 2010).

Moreover, it has been widely suggested that emotion influences the response of the motor system. Most evidence in support of this statement comes from studies that investigated the effects induced by the observation of emotional pictures upon the motor system (Bradley et al., 1993; Oliveri et al., 2003; Azevedo et al., 2005; Pereira et al., 2006; Hajcak et al., 2007; Coombes et al., 2009; Coelho et al., 2010; Borgomaneri et al., 2012, 2014; Enticott et al., 2012; Hill et al., 2013). However, such studies never measured the activity of the motor system as a real-time action directed to an emotion-laden object unfolds. Enticott et al. (2012), for instance, examined CSE while participants observed videos of a static hand or hand movements after being shown a series of emotion-laden pictures. A higher CSE was found during the observation of hand movements presented after unpleasant pictures. In this study, the hand movement was directed to a mug, i.e., an object totally unrelated to the pictures' emotional content. However, the goal of the action represents a key aspect that modulates the activity of the motor system (Koch et al., 2010; Donne et al., 2011; Rizzolatti et al., 2014; Aihara et al., 2015 for review). In a previous study, we therefore devised a set of experiments in which the activity of the motor system was assessed through a realistic experimental paradigm in which participants had to grasp an emotion-laden stimulus (de Oliveira et al., 2012; Nogueira-Campos et al., 2014). The results showed that preparing to interact with unpleasant stimuli increases the motor system activity compared to pleasant ones. Based on these findings, we suggest that an unpleasant stimulus triggers aversive-like circuits in the brain whose activity has to be overcome so that action can be implemented, whereas a pleasant stimulus facilitates action implementation (de Oliveira et al., 2012; Nogueira-Campos et al., 2014).

Since many of our interactions in the environment rely on our ability to code the actions and/or emotions of others, in this study we designed an experiment to assess the impact of observing actions directed to emotion-laden objects on the motor system. The present study focused on the CSE of the observer's motor system while they watched video-clips depicting grasping directed to emotion-laden objects. We hypothesized that observing grasping directed to emotion-laden objects should induce a specific modulation upon CSE depending on the objects' valence content—unpleasant or pleasant. Accordingly, we expected that the valence of the to-be grasped objects should be taken into account during the observation of grasping directed toward them. More specifically, CSE should be higher when observing grasping directed to unpleasant objects. Thus, reflecting the higher preparatory activity related to the observation of grasping directed to that category of the objects.

## Materials and methods

### Participants

All volunteers provided informed consent for their participation in the experiments of this study. The experimental protocols were conducted according to the Declaration of Helsinki and were approved by the local ethics committee of the Clementino Fraga Filho University Hospital at the Federal University of Rio de Janeiro (004/09). Volunteers did not present or have a personal or family history of any neurological or psychiatric disorder. Also, they were right-handed according to the Edinburgh Handedness Inventory (Oldfield, 1971).

### Selection of emotional video-clips

Sixty-five video-clips depicting the right-hand of an actor grasping different objects were used. All videos had a duration of 5 s. Movement time lasted approximately 2 s. The objects were grabbed with the index finger and the right thumb (pinch grip). Ninety healthy participants (62 women and 22 men, mean age ± SD: 21.1 ± 2.54 years) were instructed to watch each video-clip presented randomly on a screen positioned in front of them. After each video presentation, they were asked to evaluate each of them by means of the *Self-Assessment Manikin Scale* (Lang et al., 2008), as employed previously for emotional-laden stimuli (de Oliveira et al., 2012). In this affective rating scale, each video-clip was classified in their valence and arousal dimensions. Ratings of valence are indicated by the graphical representation of facial expressions ranging from a severe frown (most negative) to a broad smile (most positive). For arousal, this scale varies from a state of low to high alert. Participants may select any of the five figures, or the four blank spaces in between, on a nine-point rating scale for each dimension. In the valence dimension, nine represents the extreme of pleasantness, and one represents the extreme of unpleasantness. Likewise, for arousal, nine represents a high rating, and one represents a low rating. Upon each video-clip presentation, participants had 10 s to rate it based on these two measures. When a video-clip was rated between 4.5 and 5.5 for valence dimension with a low level of arousal (1–3) it was classified as neutral. Video-clips with lower and higher valence value with respect to the neutral set were then categorized as unpleasant and pleasant video-clips, respectively (Table 1).

**Table 1:** **Valence and arousal ratings for each video-clip**.

**Pleasant**			**Neutral**			**Unpleasant**		
**Objects**	**Valence**	**Arousal**	**Objects**	**Valence**	**Arousal**	**Objects**	**Valence**	**Arousal**
Rolled money	8.35	6.97	Television remote control	5.59	2.72	Spider	2.74	6.44
Chocolate candy	7.76	6.06	Calculator	5.39	2.78	A guava with worms	2.84	6.21
A piece of Brazilian cake	7.50	5.67	Sunglasses case	5.36	2.73	An embalmed rat	2.84	6.20
Car key	7.46	5.60	Video tape	5.34	2.70	An embalmed mouse	2.96	5.93
A can of chocolate milk	7.37	5.47	Floss box	5.34	2.70	An embalmed frog	3.09	5.63
A piece of chocolate	7.24	5.27	Ink cartridge	5.30	2.62	Artificial excrement	3.18	5.44
Packet of condom	7.14	5.12	Spool of thread#2	5.19	2.45	A piece of cake with hair	3.39	4.95
Ipod	7.14	5.11	Charger	5.10	2.31	A denture	3.47	4.79
A piece of sweet bread	7.04	4.96	Soap dish	5.10	2.30	Toast with a fly	3.51	4.70
Cell phone	6.88	4.71	A Rubber stamp	5.09	2.29	Mousetrap	3.53	4.65
Credit card	6.84	4.65	Adhesive tape	5.08	2.27	An embalmed fetal skull	3.54	4.62
Jewelry box	6.73	4.47	Spool of thread	5.05	2.22	An embalmed fetal head	3.59	4.51
Toast with cheese	6.60	4.28	Band-aid box	5.02	2.17	A pack of cigarettes	3.67	4.32
Credit card#2	6.57	4.23	Foot emery	5.00	2.14	An embalmed human eye	3.76	4.14
Computer mouse	6.35	4.34	Gate remote control	4.96	2.07	A piece of bread	3.86	3.91
Car key#2	6.22	3.70	Staples box	4.90	1.97	Kidney	3.96	3.68
Flower	6.09	3.50	Box of clips	4.83	1.86	An embalmed gizzard	4.02	3.55
Deodorant	6.09	3.49	Pencil case	4.83	1.85	An embalmed fish head	4.22	3.09
A little teddy bear	6.04	3.41	White box	4.82	1.85	Cockroach	4.37	2.77
A pack of candy	5.91	3.21				Medicine box	4.41	2.66
Soap	5.87	3.56				Kidney#2	4.42	2.65
Wristwatch	5.85	3.13						
Hairbrush	5.70	3.28						
Ball	5.62	2.76						
A guava	5.60	2.74						

A one-way Anova revealed a main effect of *valence* (neutral, pleasant, and unpleasant) [*F*_(2, 62)_ = 168.17, *p* < 0.001; $n_{p}^{2}=$ 0.84; β = 0.81]. *Post hoc* comparisons revealed that the observation of the unpleasant video-clips (mean ± SE: 3.49 ± 0.11) scored significantly lower than the neutral (5.20 ± 0.05) and the pleasant ones (6.64 ± 0.15), whereas the observation of the neutral video-clips scored significantly lower than the pleasant ones. In addition, there was a main effect for *arousal* [*F*_(2, 62)_ = 32.01, *p* < 0.001; *n*^2^_*p*_ = 0.51; β = 0.88]. *Post-hoc* comparisons revealed that the observation of the unpleasant (4.52 ± 0.26) and pleasant video-clips (4.39 ± 0.22) scored similarly in terms of arousal (*p* = 0.89), and both scored significantly higher than the neutral ones (2.32 ± 0.07, *p* < 0.01; Table 1).

**Figure 1:**
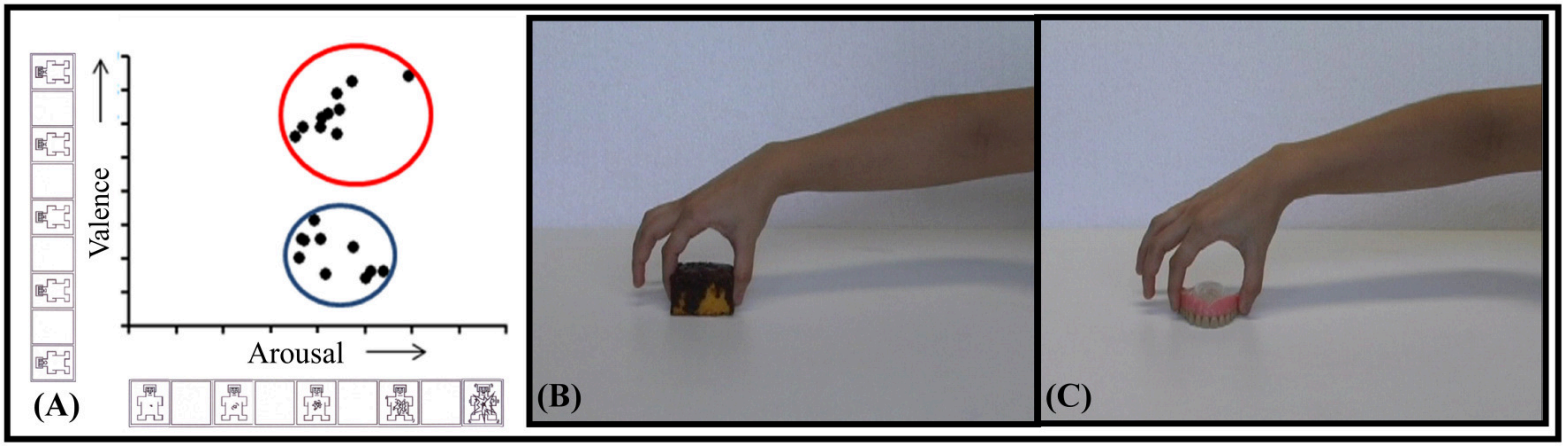
**Selected video-clips. (A)** Distribution of selected video-clips in valence and arousal dimensions. The blue circle indicates the *unpleasant*, and the red one, the *pleasant selected video-clips*. Snapshot examples of pleasant **(B)** and unpleasant **(C)** video-clips.

Ten of the video-clips classified as pleasant and 10 as unpleasant were selected to study the effect of valence on CSE during action observation (Figure 1). In terms of valence, the observation of pleasant video-clips (7.31 ± 0.15) was scored as significantly higher than that of the unpleasant (3.21 ± 0.10; *p* < 0.001; β = 0.99). In the arousal dimension, the observation of pleasant video-clips (5.38 ± 0.23) was comparable to the unpleasant ones (5.36 ± 0.23; *p* = 0.95). This precaution was taken as distinct neurobehavioral responses can be triggered depending on the arousal level for a same emotional category (Calvo and Avero, 2009; Leite et al., 2012; Wiens and Syrjänen, 2013).

In addition, the hand aperture used by the actor to grasp each object was measured for each video-clip. For this purpose, the specific frame in which the actor touched, and grabbed the object was identified by means of Movie Maker software. After that, the frame was assessed using the Irfanview program and a line between the index finger and thumb was traced to measure the distance between them. There was no significant difference in grip aperture when manipulating pleasant (5.47 ± 0.20 cm) and unpleasant (5.14 ± 0.26 cm) categories (*p* = 0.25). These objects were also balanced in weight so that pleasant (45.86 ± 3.72 g) and unpleasant (37.64 ± 3.32 g) objects did not differ (*p* = 0.20). This allowed for control of the crucial elements involved in grasping actions, since both the degree of muscle strength and the type of grasping required to manipulate the objects influence the level of recruitment of the motor system (Hendrix et al., 2009; Alaerts et al., 2010a,b).

### Procedure

**Figure 2:**
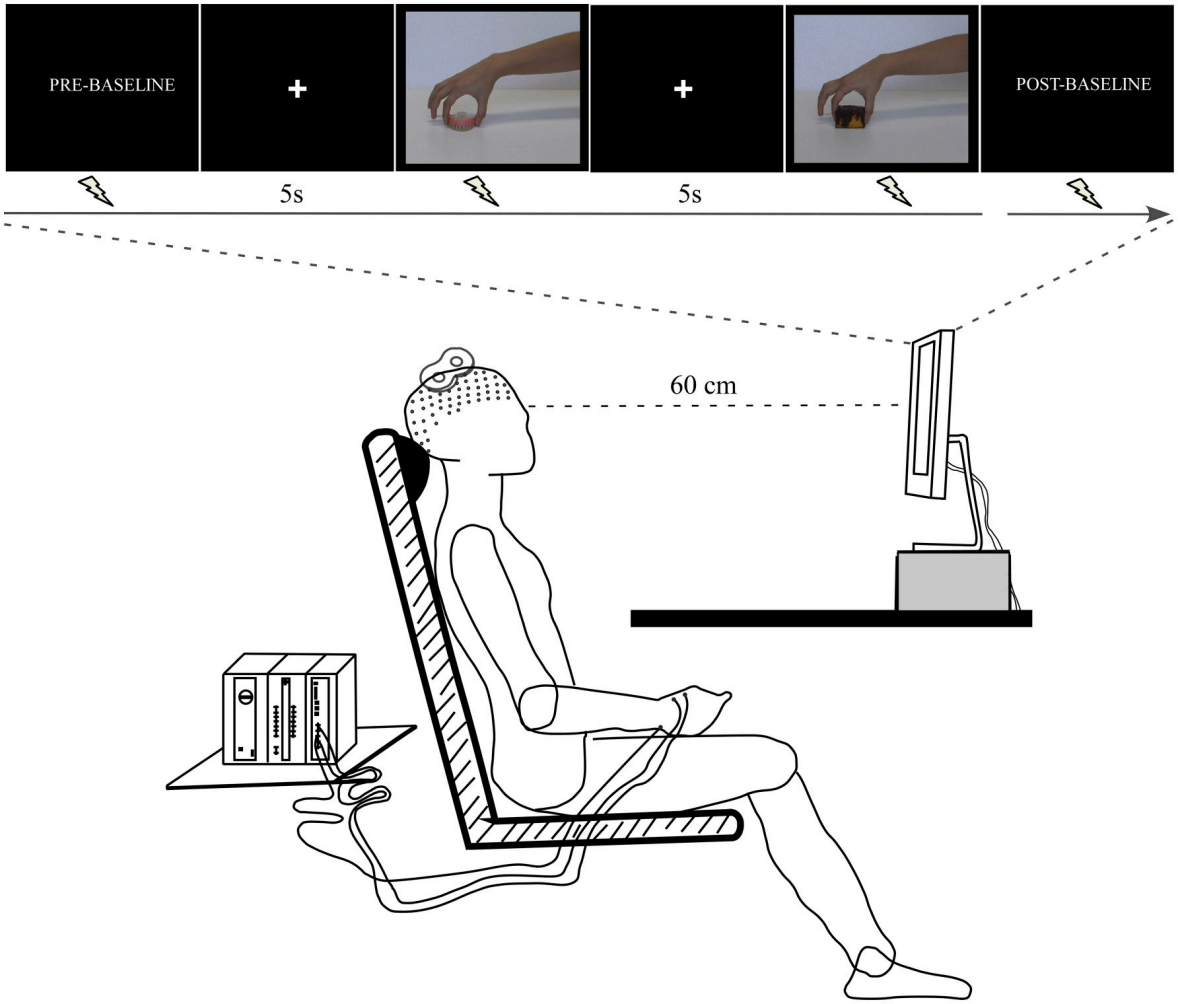
**Experimental procedure**. The participant sat at a table where a computer screen was positioned. The arms remained at rest throughout the experimental session. The TMS coil was placed over the left motor cortex. The electromyographic (EMG) signal was recorded from *right first dorsal interosseus* (FDI) muscle.

A further 14 volunteers (eight women and six men; mean age ± SD: 23.77 ± 4.75 years) were invited to passively observe the emotion-laden video-clips (pleasant and unpleasant) in order to examine the effect upon CSE. In a dimly lit room, the participants sat on a comfortable chair at a table where a 19-inch screen was positioned 60 cm away from them (Figure 2). At the beginning of the experiment, the right hand of the participant was positioned with the palm facing down over a pillow placed under the table, while the left arm was positioned over their leg. This position was kept throughout the experimental session. The experimenter read the following instructions before the experiment started: “Your task is to watch the video-clips that will be presented on the screen. These video-clips depict the hand of an actor grasping different objects. Please pay attention to them in order to answer questions at the end of the experiment. Thank you for your participation.” Then, a black screen that acted as a baseline was presented for 2 min (*Pre-Baseline*). Following this period, a white cross aligned with the center of the scene appeared on the black screen to focus the participant's gaze on this spot, and was followed by presentation of the video clips. This black screen with a white centered cross was presented for 5 s between each individual clip. A total of 10 videos of each emotional category (pleasant and unpleasant) were randomly presented twice. At the end of this period, there was another baseline period (*Post-baseline*). The above sequence comprised an experimental block. A total of two blocks were carried out. TMS pulses were applied randomly during the video-clip presentation: an equal number either at maximum *grip aperture* or *contact time*. Thus, the total number of trials per condition (*maximum grip aperture* and *contact time*) and per emotional category was 20 per participant. The pulse was applied at these two different moments based on the grasping adjustments evolving through time; i.e., the phase when the hand is open to its maximum, followed by the phase of the hand touching the object (Jeannerod, 1984). The maximum grip aperture was considered as the time (≈70% of movement duration) when the hand reached the widest grip aperture value of the index-thumb distance. In addition, TMS pulses were delivered ten times at regular intervals during *Pre-baseline* and *Post-baseline* periods. The interval between TMS pulses was approximately 9–10 s, in order to avoid cumulative effects (Chen et al., 1997; Rothwell et al., 1999). Videos were presented using the Presentation software (Neurobehavioral System, Inc., Albany, CA). Blocks were separated by 5 min of rest. During this period, instructions concerning the upcoming block were repeated. Figure 2 presents the experimental procedure.

Before the experiment started participants were exposed to a familiarization session during which they watched two video-clips from each emotional category that were not presented during the experimental session.

### Video-clip rating

The 20 video-clips presented during the TMS session were evaluated at the end of the experiment in valence and arousal dimensions by 13 participants. Upon each video-clip presentation, participants had 10 s to classify how they had felt when they observed each emotional video-clip in the affective rating scale (SAM; Lang et al., 2008) using the same procedure previously described in de Oliveira et al. (2012). The duration of the entire experimental session was around 50 min.

### Corticospinal excitability (CSE)

CSE was measured by applying single pulses of Transcranial Magnetic Stimulation (TMS) by means of a double coil powered by a Magstim stimulator (Magstim 200; Magstim Co., Whitland, UK). A cap containing a 1 cm^2^ spaced grid was positioned over the participant's skull to guide the TMS coil placement. Earplugs were provided to protect the participant's hearing. The coil was positioned tangentially over the optimal scalp location of the left primary motor cortex. First, the optimal position (hot spot) for eliciting motor-evoked potentials (MEPs) from the *right* first *dorsal interosseous* (FDI) muscle was identified. The resting motor threshold was then defined as the minimal intensity needed to evoke MEPs larger than 50 μV peak-to-peak amplitude in the FDI in at least three out of six pulses. The stimulation intensity was then set at 110% of the motor threshold to evoke MEPs.

### Electromyographic signal acquisition

The electromyographic (EMG) signal was recorded using two pairs of Ag-AgCl electrodes, arranged in a bipolar montage over the belly of the *right* FDI. EMG activity was recorded using an EMG100 acquisition module coupled to an MP150 amplifier (BIOPAC Systems Inc., USA) and stored on a computer for offline analysis. Data were sampled at 20 KHz and band-pass filtered between 10 and 5 KHz with a 60 Hz notch filter.

### Data analysis

MEPs were quantified based on their latency and peak-to-peak amplitudes using a MATLAB routine (Mathworks, USA). This routine was designed to segment the EMG epochs corresponding to each trial. The beginning and the end of each MEP were marked manually on each trial. The latency was computed by counting the time elapsed between the TMS trigger and the beginning of the MEP response in the EMG signal. The MEP amplitude was calculated by measuring the peak-to-peak amplitude. The root-mean-square (RMS) of the EMG activity 200 ms prior to the TMS pulse was measured to ensure that the EMG baseline activity remained lower than 10 μV for all experimental conditions.

Outlier detection was computed by calculating the mean latency and mean MEP amplitude for each specific block and each participant. Latency and MEP amplitude values exceeding 2.5 standard deviations from the mean were marked as outliers and discarded. Based on this criterion, 10% of the trials were discarded from the analyses. The number of discarded trials did not differ between emotional categories (*p* = 0.79). Given that the CSE did not change between *Pre-baseline* (0.87 μV ± 0.64) and *Post-baseline* (0.92 μV ± 0.54; *p* = 0.67), these measures were collapsed into one baseline condition. The MEP amplitudes collected during emotional video-clips were normalized relative to this baseline for each participant within the block.

### Statistical analysis

Statistical analysis was performed with SPSS (SPSS; San Rafael, CA). A three-way repeated-measures Anova was used to compare CSE based on *valence* (pleasant and unpleasant), *conditions* (maximum grip aperture and contact time), and *blocks* (1 and 2). Tests of normality were performed to determine the probability that the sample came from a normally distributed population (Shapiro-Wilk's W test, *p* ≥0.05). Data sphericity was verified before each test (for all tests: *p* ≥0.05). The level of significance was set to 0.05. Tukey HSD *post-hoc* analysis was employed to test individual comparisons whenever a statistical significance was attained. *T*-test was used for comparing the video-clip ratings on valence and arousal dimensions. The effect size was computed based on the partial eta-squared (*n*^2^_*p*_). Also, the statistical power (β) was indicated whenever applicable.

### Results

#### Video-clip rating

**Figure 3:**
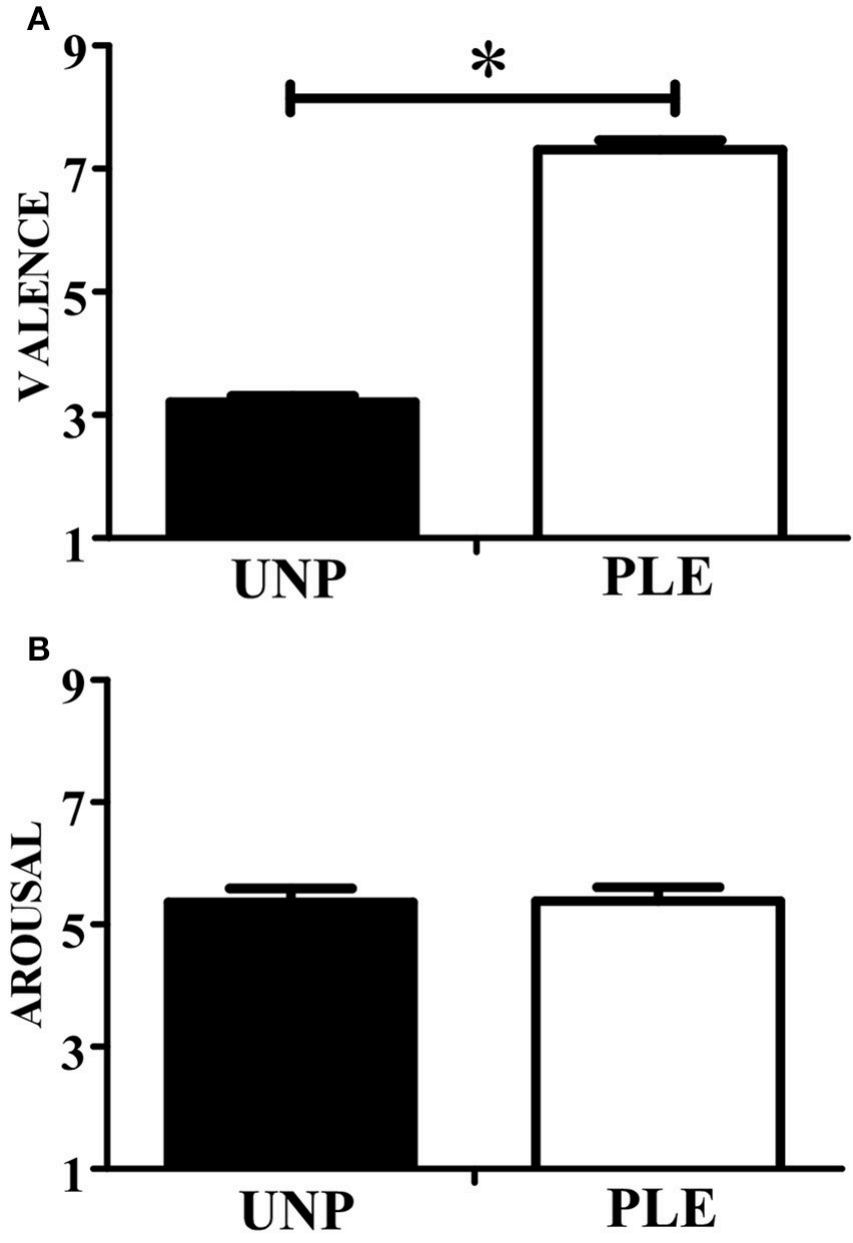
**Video-clip rating. (A)** Scores for the valence dimension. **(B)** Scores for the arousal dimension. UNP, unpleasant and PLE, pleasant (^*^p < 0.05).

In terms of valence, the observation of unpleasant actions (2.85 ± 0.25) scored significantly lower than that of pleasant actions (6.81 ± 0.22; *p* ≤ 0.01; β = 0.99). In addition, the observation of unpleasant (4.43 ± 0.45) and pleasant videos (3.78 ± 0.49) scored similarly in terms of *arousal* (*p* = 0.22). These results can be seen in Figure 3.

#### Corticospinal excitability (CSE)

**Figure 4:**
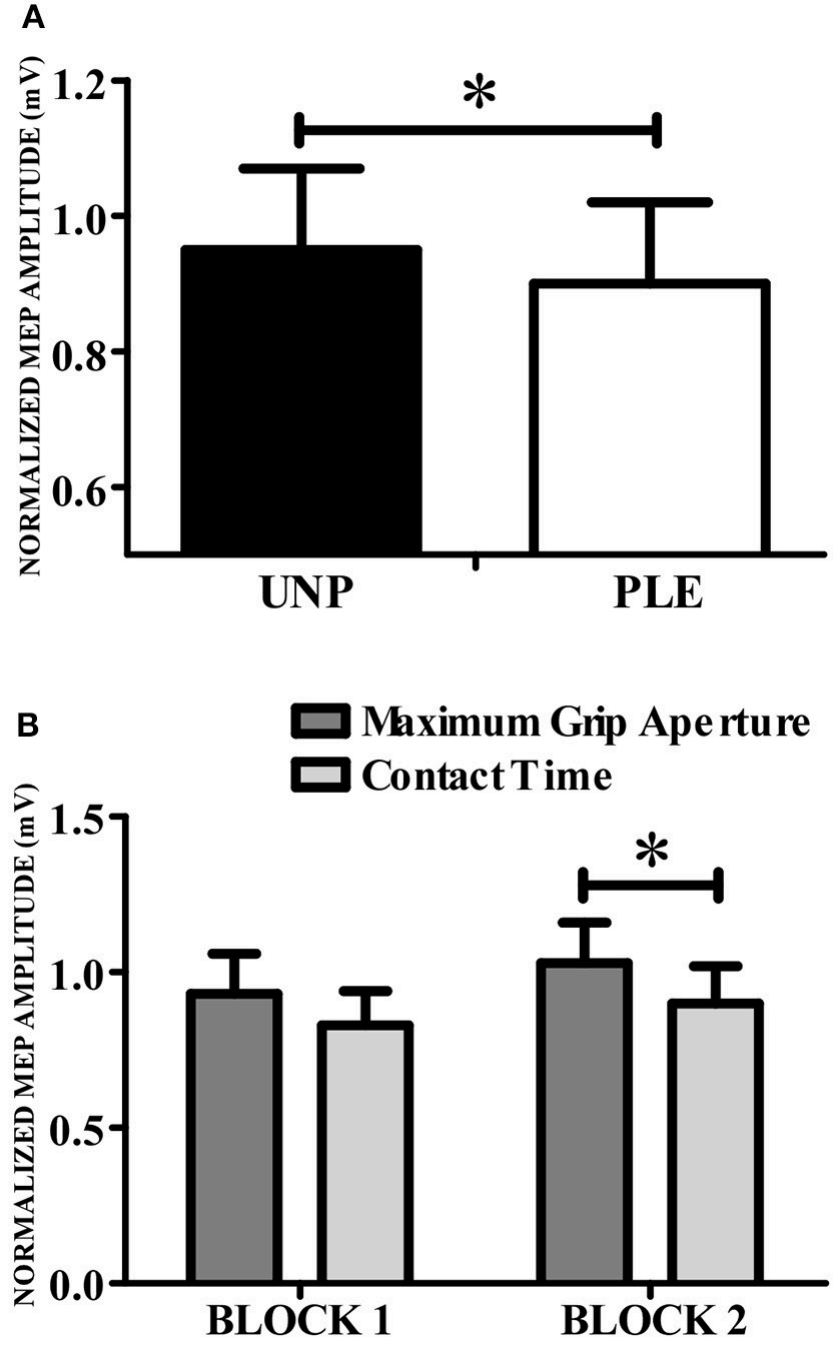
**Corticospinal excitability. (A)** CSE was higher during the observation of grasping directed to unpleasant (black bars) compared to pleasant (white bars) objects. **(B)** CSE was higher for grip aperture than for contact time. UNP, unpleasant; PLE, pleasant (^*^p < 0.05).

A repeated-measures Anova revealed a main effect of valence [*F*_(1, 13)_ = 102.57, *p* = 0.007; *n*^2^_*p*_ = 0.44; β = 0.84], indicating that CSE was higher during the observation of grasping unpleasant (0.95 ± 0.12) compared to pleasant (0.90 ± 0.12) objects (Figure 4A). This analysis also resulted in a significant condition vs. block interaction [*F*_(1, 13)_ = 7,34, *p* = 0.02; *n*^2^_*p*_ = 0.36; β = 0.71]. *Post hoc* analysis showed that CSE was higher during the observation of maximum grip aperture (0.98 ± 0.12) compared to contact time (0.87 ± 0.12) during block 2 (Figure 4B). There was a tendency for condition [*F*_(1, 13)_ = 4,23, *p* = 0.06; *n*^2^_*p*_ = 0.25; β = 0.48], but neither other main effects nor any significant interactions (see Table 2).

**Table 2:** **MEP values (mV) per experimental condition**.

	**Mean**	**Standard Error**
**VALENCE**		
**Unpleasant**	0.945	0.121
**Pleasant**	0.896	0.124
[*F*_(1. 13)_ = 102.57. *p* = 0.007; *n*^2^_*P*_ = 0.44; β = 0.84]		
**MAXIMUM GRIP APERTURE**		
**Block 1**	0.926	0.126
**Block 2**	1.027	0.125
**CONTACT TIME**		
**Block 1**	0.826	0.113
**Block 2**	0.902	0.124
[*F*_(1. 13)_ = 7.34. *p* = 0.02; *n*^2^_*p*_ = 0.36; β = 0.71]		

## Discussion

This aim of the study was to evaluate the effect of emotion on the motor system when the goal of the action was to interact with the source of the emotion. An ensemble of objects was selected and video-clips that mimic grasping actions in the real world were made. These videos were categorized using the Self-Assessment Manikin (Lang et al., 2008) and unpleasant and pleasant video-clips differing in *valence* but not in *arousal* were selected. To test the effect on CSE of passive observation of grasping actions directed to emotion-laden objects, TMS pulses were applied during presentation of the videos either at the moment of *maximum grip aperture* or *contact*. CSE was higher during the observation of grasping directed to unpleasant compared to pleasant objects. In addition, a larger CSE was found at the moment of maximum grip aperture compared to the moment of contact.

Unlike previous studies that investigated the effect of emotion over the motor system through the observation of emotional-laden pictures (Oliveri et al., 2003; Hajcak et al., 2007; Coelho et al., 2010; Enticott et al., 2012; Hill et al., 2013), in the present study videos depicting a goal-directed action were used. The observation of actions directed to an object provides a way to study the motor representations enrolled in the action itself (Koch et al., 2010). In addition, when observing an action, the target of the action seems to be taken into account (Fogassi et al., 2001; Umiltà et al., 2001; Cattaneo et al., 2005, 2009; Koch et al., 2010; Ocampo and Kritikos, 2011). This is in agreement with the basic idea that the motor system represents the transformations of goal-relevant sensory information to code motor outputs (Johansson and Cole, 1992). Herein, the higher CSE prompted by the observation of grasping directed to unpleasant compared to pleasant objects indicates that the valence implied in the actions' goal influences the observer's motor representations. Indeed, the observation of an action seems to automatically retrieve its motor representations (Rizzolatti et al., 1988, 2014; Rizzolatti and Craighero, 2004).

Notably, the CSE modulation during action observation matches the effects of valence found during motor preparation when actually grasping objects. In previous studies we examined the effects of preparing to grasp emotion-laden stimuli on readiness potential (RP; de Oliveira et al., 2012) and on CSE (Nogueira-Campos et al., 2014). RP is a marker of motor preparation and reflects the recruitment of the fronto-parietal areas preceding a voluntary movement (Shibasaki and Hallett, 2006). The CSE prompted by applying a TMS pulse over the primary motor cortex before the movement onset reflects preparatory activity as well (Hasbroucq et al., 1997). We found higher RP preceding grasping directed to unpleasant stimuli and lower RP directed to pleasant ones (de Oliveira et al., 2012). Likewise, we found a higher CSE for unpleasant stimuli and a lower CSE for pleasant ones when the TMS pulse was applied before the movement onset (Nogueira-Campos et al., 2014). The CSE seemed to reflect the higher recruitment of motor-related areas when the participants prepared to act in the unpleasant as compared to the pleasant category. Hence, when participants are asked to interact with emotion-laden stimuli they estimate the value embedded in the action's goal.

These changes not only occur when participants are preparing to grasp objects but are also triggered when participants observe others' actions, and unfold over time (Gangitano et al., 2004), giving support to the idea that during action observation the observer anticipates the outcome of others' actions (Kilner et al., 2004; Neal and Kilner, 2010; Rizzolatti et al., 2014). Thus, observing an action directed to emotion laden-objects may have triggered the motor representations in a predictive way, leading to a valence-laden modulation over the CSE in accordance with the effects that have previously been described during motor preparation (de Oliveira et al., 2012; Nogueira-Campos et al., 2014).

As expected, the observation of grasping directed to emotion-laden objects also prompted a higher CSE at the moment of maximum grip aperture compared to the moment of contact. Indeed, CSE is modulated based on the mechanical changes of the hand, i.e., higher for maximum grip aperture during the observation of reach-to-grasp actions (Gangitano et al., 2001, 2004). Herein, the coding of temporal hand adjustments was more pronounced in the second block, although there was a clear tendency in the same direction as the first block. The processing of motor cues imprinted in the observed action suggests the enrolling of the observers' motor system in coding such action, being more evident when the context is totally predictable (Kilner et al., 2004). Likewise, our results suggest that, beyond motor representations, the motor system also encodes the emotion content behind the observed action in order to guide the individuals' actions in interactive contexts. Crucially, the effect of emotion upon CSE was pervasive, possibly reflecting the core survival function of emotion (Mourão-Miranda et al., 2003; Lang and Bradley, 2010; Filmer and Monsell, 2013; Borgomaneri et al., 2014).

One could claim that the emotion-related effects on CSE are merely due to the observation of emotion-laden objects. Although there is evidence that arousal (Hajcak et al., 2007; Borgomaneri et al., 2012; Hill et al., 2013) and valence (Coelho et al., 2010; Enticott et al., 2012) of emotion-laden pictures modulate CSE, in our previous work, the observation of static graspable emotion laden-stimuli did not induce a specific modulation over the CSE (Nogueira-Campos et al., 2014). The divergent valence effect occurred only when the participants were engaged in preparing to make a movement. Such results strengthen the premise that the valence effect described here associates with the recruitment of motor representations enrolled in the preparation of the observed action itself. In addition, the present findings add to the previous one by showing a specific valence modulation over CSE during the observation of an action whose goal is to interact with the source of emotion.

On the other hand, we cannot preclude the possibility that the valence modulation over CSE is due to the recruitment of other brain regions besides the primary motor cortex. Indeed, the interactions between motor areas (putamen, pre-motor, and intraparietal cortex) and circuits coding emotion (insula, amygdala, and cingulate cortex) have been posed as fundamental in the processing of actions embedded in an emotional context (Grosbras and Paus, 2006; Pereira et al., 2010; Coombes et al., 2012). Interestingly, recent findings have proposed the insula, a region traditionally related to emotion expression (Bechara and Naqvi, 2004; Craig, 2009), as central for modulating motor system activity during the observation of arm movements (Di Cesare et al., 2015). Further studies should be conducted to broaden the investigation about the role of the motor system, including the action-perception network, during the action directed to emotional-laden objects.

Finally, the present findings indicate that the valence implied in an observed action goal prevails over motor representations. Taken together, these results corroborate the proposal that both the temporal dynamics as well as the action goal are taken into account by the motor system during grasping directed to an emotion-laden object. The privileged influence of valence over CSE can reflect the capacity of the motor system to predict the consequences of actions in emotional interactive contexts. Further, this capacity may be crucial in correctly responding to other's actions.

## Author contributions

Conception and design of the work: AN, GS, VD, LD, ER, and CV. Performing the experiments: AN and GS. Data Analyses: AN, VD, and CV. Writing the paper: AN and CV.

## Funding

This work was supported by Conselho Nacional de Desenvolvimento Científico e Tecnológico (CNPq), Programa Pós-doutorado Júnior (PDJ; Process number: 159974/2012-7), CNPq (grant numbers 306817/2014-4 and 480108/2012-9), Fundação de Amparo a Pesquisa do Estado do Rio de Janeiro FAPERJ (grants E-26/110.526/2012; E-26/010.002902/2014) and Financiadora de Estudos e projetos FINEP (PROINFRA HOSPITALAR grant 18.569-8). This research has been conducted as part of the activities of the FAPESP Research, Dissemination and Innovation Center for Neuromathematics-NeuroMat (FAPESP, grant 2013/07699-0). This work was also supported by CAPES-COFECUB (Project no. 819-14).

### Conflict of interest statement

The authors declare that the research was conducted in the absence of any commercial or financial relationships that could be construed as a potential conflict of interest. The reviewer OG and handling Editor declared their shared affiliation, and the handling Editor states that the process nevertheless met the standards of a fair and objective review.
